# Marine bacteria degrade viral particles as a source of nitrogen, phosphorus, and sulfur-rich dissolved organic matter

**DOI:** 10.1093/ismeco/ycag056

**Published:** 2026-03-09

**Authors:** Hongcong Man, Xiaojue Li, Jihua Liu, Chen He, Quan Shi, Xilin Xiao, Wei Wei, Feng Chen, Yongle Xu

**Affiliations:** Institute of Marine Science and Technology, Shandong University, Qingdao 266237, China; Shandong Key Laboratory of Intelligent Marine Engineering Geology, Environment and Equipment, Qingdao 266237, China; Institute of Marine Science and Technology, Shandong University, Qingdao 266237, China; Shandong Key Laboratory of Intelligent Marine Engineering Geology, Environment and Equipment, Qingdao 266237, China; Institute of Marine Science and Technology, Shandong University, Qingdao 266237, China; Shandong Key Laboratory of Intelligent Marine Engineering Geology, Environment and Equipment, Qingdao 266237, China; State Key Laboratory of Heavy Oil Processing, China University of Petroleum, Beijing 102249, China; State Key Laboratory of Heavy Oil Processing, China University of Petroleum, Beijing 102249, China; Innovation Research Center for Carbon Neutralization, Xiamen University, Xiamen 361002, China; Hubei Key Laboratory of Microbial Transformation and Regulation of Biogenic Elements in the Middle Reaches of the Yangtze River, State Key Laboratory of Green and Efficient Development of Phosphorus Resources, School of Environmental Ecology and Biological Engineering, Wuhan Institute of Technology, Wuhan 430205, China; Institute of Marine and Environmental Technology, University of Maryland Center for Environmental Science, Baltimore, MD 21202, United States; Institute of Marine Science and Technology, Shandong University, Qingdao 266237, China; Shandong Key Laboratory of Intelligent Marine Engineering Geology, Environment and Equipment, Qingdao 266237, China

**Keywords:** viruses, DOM, virion DOM, bacterial degradation, FT-ICR MS

## Abstract

Viral particles are abundant in aquatic and soil environments and are operationally defined as part of dissolved organic matter (DOM) in nature. Virions are known to contain rich nitrogen, phosphorus, and sulfur, and they represent a special pool of DOM. Little is known about whether bacteria can use virion DOM to support their growth. In this study, we added purified phage particles to three non-host marine bacterial cultures to investigate how marine bacteria respond to the addition of purified virions. Upon adding virions, we monitored bacterial and viral abundance, bacterial extracellular enzymatic activities, and the composition of virion DOM. Bacterial growth increased with the addition of purified virions, and a large portion of the virions was degraded in the three bacterial cultures within 6 days of incubation. High activities of exocellular alkaline phosphatase and leucine aminopeptidase suggest these exoenzymes are involved in virion degradation. Using an ultra-high-resolution mass spectrometer, we obtained the first van Krevelen diagram of virion DOM molecules. A total of 508 DOM molecules derived from virions were detected, and 77 virion DOM molecules shared across all three bacterial cultures were not detected after 6 days. Together, our results show that bacteria tend to utilize nitrogen-, phosphorus-, and sulfur-rich DOM molecules resulting from virions. This study provides direct evidence that purified virions can serve as a DOM source to support bacterial growth, implying that viruses in the natural environment are a unique source of DOM, which would exert profound impacts on marine food webs and biogenic element cycling.

## Introduction

Viruses are abundant and play an important role in regulating microbial population dynamics and biogeochemical cycles in the ocean [1–3]. Lytic infections of viruses result in cell lysis and the release of cellular components of the host. In the laboratory study, the term “lysate” is often used to describe the entirety of viral lysis products, including dissolved organic matter (DOM), inorganic elements, viral particles, cell wall debris, etc. Extensive studies have been conducted to understand the impact of viral lysis on marine carbon cycling. “Viral shunt” has been used to emphasize the trophic flow of organic matter and nutrients resulting from viral infection and lysis [4, 5]. Viral lysates can alter the composition of marine DOM [6–10]. Compared to cell exudation and mechanical cell lysates, viral lysates are rich in sulfur and nitrogen [8–10] and contain elevated levels of amino acids [11, 12], urea [11], and protein-like components [7, 13], which makes them more available to microbial communities [7, 9, 10]. It has been reported that viral lysates can promote the growth of bacterial isolates [14–16] and prokaryotic communities [17–25]. Viral lysates of picocyanobacteria not only stimulate rapid growth of heterotrophic bacteria but also alter their community composition [24, 25]. Viral lysates contain a complex organic mixture including viral particles. It is still unclear whether viral particles alone can contribute to bacterial growth, and if so, to what extent and in what forms this utilization occurs.

Viral particles are composed primarily of nucleic acids and proteins, biomolecules that are rich in carbon, nitrogen, and phosphorus [26–30]. When lytic viruses enter bacterial cells, they hijack the host replication system to replicate phage genome and produce proteins (and sometimes lipids) needed to package phages by reprogramming the hosts' metabolism, such as increasing carbon absorption, reducing carbon fixation, and promoting nitrogen absorption [31–40]. Typically, the burst size of bacteriophage ranges from 50 to 100 [41–44], meaning that upon successful infection, one phage can produce 50–100 new phage particles. Consequently, viruses released into the environment are primarily composed of nucleic acids (either DNA or RNA) and proteins (capsids), occasionally lipid membrane. These components—nucleotides, proteins, and lipids—together contain the essential elements of carbon, hydrogen, oxygen, nitrogen, phosphorus, and sulfur. It seems plausible that viral particles contain complex organic molecules elements that can be used to support bacterial growth.

The vast majority of viral particles in the natural environment are small enough to be considered as DOM (<0.45 μm). Viruses contribute substantially to marine DOM due to their high abundance in seawater [1, 5, 45, 46]. Bacteriophages are the dominant form of viruses in seawater [46, 47], and can serve as a potential nutrient source for other organisms [45, 48]. It is reported that protozoa (e.g. flagellates, ciliates) and certain zooplankton can graze viral particles, transferring their nutrients into higher trophic levels (classic food webs) [41, 46, 49–51]. Inactivated virions treated with freeze–thaw and microwave methods can promote the growth of the prokaryotic community in sediments [52]. When ^33^P-labeled viral lysates (virions and cell debris, 0.2 μm-filtered) were added to bacterial communities from oligotrophic seawater, 50% of ^33^P was retained by 0.2 μm filters within 7 h, implying that virions may be taken up by the bacterial communities as a source of DOM [53]. Furthermore, protease activity in marine sediments correlates positively with viral decay rates, suggesting enzymatic reactions facilitate bacterial breakdown of virions by providing potential substrates such as polypeptides and nucleic acids [52, 53]. It has been estimated that bacteria can consume 0.1%–4% of DOC per day via nanoparticles like viruses [54]. Although earlier studies suggest that phage particles may be utilized by bacteria as a source of DOM to support their growth, no direct evidence is available to confirm that purified phage particles (as opposed to whole-cell lysate) can support bacterial growth.

In this study, we employed the ultracentrifugation-purified virions (roseophage RDCBphi1) as the sole organic substrate to grow three representative marine bacterial strains, *Ruegeria pomeroyi* DSS-3, *Alteromonas macleodii* ATCC 27126, and *Arenibacter* CBW1107-13. They represent bacteria from *alphaproteobacteria*, *gammaproteobacteria*, and *Flavobacteriia*, respectively. Following the addition of purified virions, we analyzed bacterial growth, extracellular enzyme activities, and DOM molecules (based on ultrahigh-resolution Fourier transform ion cyclotron resonance mass spectrometry (FT-ICR MS)). We attempted to investigate: (i) whether purified virions can support bacterial growth; (ii) if so, how fast virions will be degraded by bacteria; (iii) how bacterial enzymes respond to the addition of purified virions; (iv) how the virion DOM composition change after being added into bacterial cultures.

## Materials and methods

### Experimental setup and sample collection

Roseophage RDCBPhi1 (GenBank accession no. PX583097) and three bacterial strains (*R. pomeroyi* DSS-3, *A. macleodii* ATCC 27126, and *Arenibacter* CBW1107-13) were used to set up the experiment. Purified RDCBPhi1 suspensions were obtained using the method described by Zheng et al. [55]. Briefly, RDCBPhi1 was amplified by adding phage suspensions to a batch of exponentially growing host cultures (OD_600_ = 0.2) in 2-L flasks (total volume 60 L) at a multiplicity of infection (MOI) of 0.01 and incubated until lysis. RNase A and DNase I were added to the lysates at final concentrations of 2 mg L^−1^ and incubated at room temperature for 1 h. Afterward, the NaCl concentration in the lysates was adjusted to 1 M, and the lysates were incubated on ice for 0.5 h. The phage lysates were then centrifuged at 10 000 × g, 4°C for 15 min, and the supernatants were collected and filtered through 0.45-μm filters (Millipore, Bedford, MA, USA) to remove remaining debris. The filtrates were then treated with polyethylene glycol 8000 at a final concentration of 100 g L^−1^ and incubated overnight at 4°C. Phage particles were then precipitated by centrifugation at 12000 × g, 4°C for 1 h and resuspended with TM buffer (20 mM Tris-Cl and 10 mM MgSO_4_). Phage particles were then purified by ultracentrifugation in CsCl density gradients at 200 000 × g, 4°C for 8 h in a SW 41 Ti rotor (Beckman Optima L-100XP, Beckman Coulter, CA, USA). Visible viral bands were extracted. CsCl in the extracted viral band fraction was removed using 30-kDa cutoff centrifugal ultrafiltration units (Millipore, Bedford, MA, USA) with autoclaved seawater (20 ppt) or Milli-Q water, depending on the subsequent use for bacterial incubation or FT-ICR MS analysis of virion DOM molecular composition. Purified phage suspensions were stored at 4°C until further use.

DSS-3, ATCC 27126, and CBW1107-13 were first cultured in rich organic (RO) medium [56] with a salinity of 20 ppt at 28°C. For each bacterial strain, 3 ml of the exponentially growing cultures (OD_600_ = 0.6) were centrifuged at 6000 × g for 10 min to remove RO medium. After being washed twice and resuspended in sterile seawater (20 ppt), bacterial cells of each strain were split equally into three flasks, with each containing 200 ml of autoclaved seawater (20 ppt), trace metals (formulated as those in the RO medium) and purified phage suspensions (Supplementary Fig. 1) at a final concentration of 1.6 × 10^11^ particles ml ^−1^. All flasks were incubated at 28°C in a shaker at a speed of 160 rpm in dark. Subsamples were collected to measure bacterial and viral abundances, exoenzyme activity, plaque forming unit (PFU), and DOM molecular composition. Specifically, subsamples for bacterial and viral abundances, exoenzyme concentrations were taken daily over an 8-day incubation; PFU samples were collected on days 0 and 6; subsamples for DOM molecular composition were collected on day 6. Additionally, bacterial growth of three strains in autoclaved seawater (20 ppt) supplemented with trace metals (formulated as those in the RO medium) was also monitored as a control. For each strain, bacterial abundance samples were taken daily over an 8-day incubation.

For microbial abundance counting, 1 ml of each culture was taken and fixed with glutaraldehyde at a final concentration of 1% v/v for 15 min in the dark. After snap freezing in liquid nitrogen, the abundance samples were kept at −80°C until flow cytometry analyses. Additionally, 2 ml of each bacterial culture was collected for PFU calculation. Exoenzyme activity samples were taken by collecting filtrates of 10 ml bacterial cultures through 0.22-μm filters (Millipore). To collect DOM in bacterial cultures, 50 ml was taken from each carboy. The three replicate subsamples for each strain were pooled together and then filtered through pre-combusted (450°C, 4 h) Whatman GF/F filters. All glassware used in this study was acid washed, rinsed with Milli-Q water, and pre-combusted (450°C, 4 h) before use.

### Bacterial and viral abundance

The bacterial and viral abundances were calculated using flow cytometer [56–58]. Briefly, the frozen samples were thawed at 37°C and diluted with Tris-EDTA buffer (pH = 8, Sigma-Aldrich, Darmstadt, Germany) to an appropriate concentration for flow cytometry analyses. The bacterial abundance samples were stained with SYBR Green I (Invitrogen) at a final concentration of 0.01% v/v for 15 min in the dark at room temperature and then analyzed by a BD Accuri™ C6 Plus Flow cytometer (Becton Dickinson, San Jose, CA, USA) [57, 58]. The phage abundance samples were stained with SYBR Green I (Invitrogen) at a final concentration of 0.005% v/v for 10 min at 80°C in the dark and cooled at room temperature for 5 min and then analyzed by a CytoFLEX Flow cytometer (Beckman Coulter, Brea, CA) [58, 59]. A Spearman correlation analysis was performed between the abundances of bacteria and viruses using the OmicStudio tools at https://www.omicstudio.cn.

### PFU determination

PFU in the bacterial cultures was analyzed to indicate the variation of active phage particles during the incubation. Briefly, the ultracentrifugation-purified phage suspension used as the initial DOM substrate for bacterial incubation was serially diluted with TM buffer. Subsamples of bacterial cultures collected on day 6 were first filtered through 0.45-μm-pore-size filters (Millipore), and the filtrates were then similarly diluted with TM buffer to appropriate concentrations. PFU was determined using the plaque assay method. One hundred microliters of each diluted phage suspension were inoculated into 900 μL of exponentially growing host cells (OD_600_ = 0.2). Following absorption for 20 min, the samples were mixed with 5 ml of RO medium with 0.5% agar (40°C) and poured onto solid RO medium with 1.5% agar in petri dishes.

### TEM observation

The purity of virion suspensions obtained by ultracentrifugation was determined using TEM observation. A drop of phage suspensions was adsorbed on carbon-coated copper grids for 10 min and then stained with 1% phosphotungstic acid for 1 min. The sample was dried for 10 min and then examined using a Tecnai G2 F20 Spirit BioTwin transmission electron microscope (FEI, Thermo Fisher Scientific, USA).

### Dissolved extracellular enzymatic activity

The activities of alkaline phosphatase (APase), β-glucosidase (BGase), and leucine aminopeptidase (LAPase) were measured as they represent the key enzymes in acquisition of phosphorus, carbon, and nitrogen, respectively, in marine bacteria [60–63]. These enzymatic activities reflect the potential degradation of protein- and nucleic acid-rich viral particles. The hydrolysis of the fluorogenic substrate analogues 4-methylumbelliferyl (MUF)-phosphatase, MUF-β-D-glucopyranoside, and L-leucine-7-amido-4-methylcoumarin (AMC) was analyzed to estimate the potential activity of APase, BGase, and LAPase, respectively [64, 65]. Briefly, in Corning 96-well black plates (Corning, USA), 150 μL of a 0.22-μm filtered subsample was incubated with 50 μL of the respective substrate at a final concentration of 250 μM for 3 h in the dark at 25°C. Fluorescence was recorded on a Biotek Cytation 5 and analyzed using Bioteck Gen 5 data analysis software (BioTek, USA) at excitation/emission wavelengths of 365/445 nm before and after the 3-h incubation [62]. The increase in fluorescence after the 3-h incubation was transformed into enzymatic activity using a standard curve established with different concentrations of the MUF and AMC added to 0.22-μm (Millipore) filtered sterile seawater. A Spearman correlation analysis was performed between the microbial abundances and the activities of APase and LAPase using the OmicStudio tools at https://www.omicstudio.cn.

### DOM molecular composition analysis using FT-ICR MS

DOM molecular composition of purified viral particles suspended in Milli-Q water and of DOM generated after bacterial growth for 6 d was determined using FT-ICR MS analysis. It is important to note that the “virion DOM” analyzed here operationally refers to the organic molecules that are derived from viral particles. A total of 10^13^ purified phage particles suspended in 150 ml of Milli-Q water and 150 ml of GF/F-filtrates of each bacterial cultures collected on day 6 were used to prepare DOM samples for FT-ICR MS analysis. DOM in the purified phage suspensions and GF/F-filtrates of each bacterial cultures were extracted by solid-phase extraction (SPE) using styrene divinyl benzene polymer cartridges (Agilent Bond Elut PPL, 500 mg) as described in [66]. Prior to extraction, each cartridge was pre-conditioned sequentially with 6 ml of methanol (Supelco) and 6 ml of 0.1% (v/v) formic acid (98%, CNW, Germany) in Milli-Q water. For extraction, each sample was acidified to pH = 2 with formic acid and then loaded onto the pre-conditioned cartridge. After sample loading, the cartridge was rinsed with 12 ml of 0.1% formic acid solution to remove salts and was subsequently dried with pure nitrogen. The DOM was eluted with 4 ml of methanol, using 1 ml per step. The entire eluate was completely transferred and collected into a pre-combusted (450°C, 4 h) amber glass vial. The methanolic extract was stored at −20°C until FT-ICR MS analysis. Specifically, virion DOM analysis targeted molecules mainly resulting from acid-disassembled virions and extractable by SPE. The FT-ICR MS analysis was conducted using a 9.4 T Apex Ultra FT-ICR MS instrument (Bruker Daltonics) featuring negative ion electrospray ionization (ESI) with a flow rate of 250 μL h^−1^ as previously described [67, 68]. The mass spectrometer was initially calibrated using sodium formate and then recalibrated with a known mass series in the SRFA (Suwannee River Fulvic Acid), which contains a relatively high abundance of CHO molecules, ranging from 200 to 800 m/z unit; and their peak intensities were exported to a spreadsheet. Data was processed with an in-house software [69]. A molecular formula calculator generated matching formulas according to the elemental combinations of ^12^C_1–60_, ^1^H_1–120_, ^14^N_0–3_, ^16^O_0–30_, and ^32^S_0–1_, with a mass accuracy window setting as 1.0 ppm and a signal-to-noise (S/N) ratio of ≥4 for formula assignment. All candidate formulas were subjected to basic chemical criteria as described by Kujawinski et al. [70]: (i) the number of H atoms must be at least one-third that of C atoms and must not exceed 2C + N + 2; (ii) the sum number of N and H atoms must be even; and (iii) the H/C and O/C values were restricted to below 3 and 1.5, respectively.

## Results and discussion

### Virion-based medium supported bacterial growth

**Figure 1:**
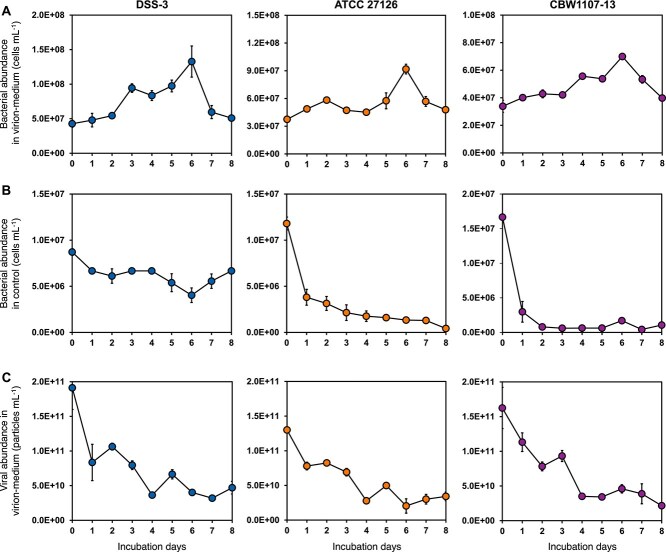
Variations of bacterial and viral abundance during the incubation. The bacterial abundance in three bacterial cultures (DSS-3, ATCC 27126 and CBW1107-13) with purified virions (A) or without purified virions (B), and viral abundance after being added into three different bacterial  cultures (C).

As the sole DOM substrate, roseophage RDCBphi1 particles supported the growth of DSS-3, ATCC 27126, and CBW1107-13, with peak bacterial abundances observed on day 6 (Fig. 1A). In contrast, none of the three strains grew in seawater without virion addition (Fig. 1B), indicating that bacterial growth with purified virions was mainly supported by the organic source of virial particles. Viral abundance showed a significant inverse trend compared to bacterial abundance (Supplementary Fig. 2), decreasing over the incubation period and reaching the lowest levels by day 6 (Fig. 1C). The viral abundance was reduced by 79.9%–84.3% on day 6 in three bacterial cultures. This decline, coupled with a consistent reduction in phage infective units (Supplementary Fig. 3), further suggests that bacteria can break down viral particles and use virion DOM to support their growth. Although abiotic degradation of viral particles may also occur, the tight coupling between bacterial proliferation and virion loss points to bacterial activity as the dominant driver of virion degradation in our system.

To assess whether the observed viral decay could stoichiometrically support the measured bacterial growth, we conducted a theoretical mass balance calculation. Assuming a viral C:N:P ratio of 19.5:6.49:1 and a bacterial C:N:P ratio of 69:16:1 [2, 6, 71], with 0.1 fg C per viral particle and 20 fg C per cell [2, 6, 71], the elements released from the phage particles (1.1 × 10^11^–1.4 × 10^11^ particles ml^−1^) were estimated to be 6.1–82.0 times the amount required to support the observed bacterial increase (3.4 × 10^7^–9.0 × 10^7^ cells ml^−1^). This indicates that DOM released from the degradation of viruses can fully support the carbon, nitrogen, and phosphorus requirements for the three bacterial growth in this study.

### Extracellular enzyme activities in virion-based cultures

**Figure 2:**
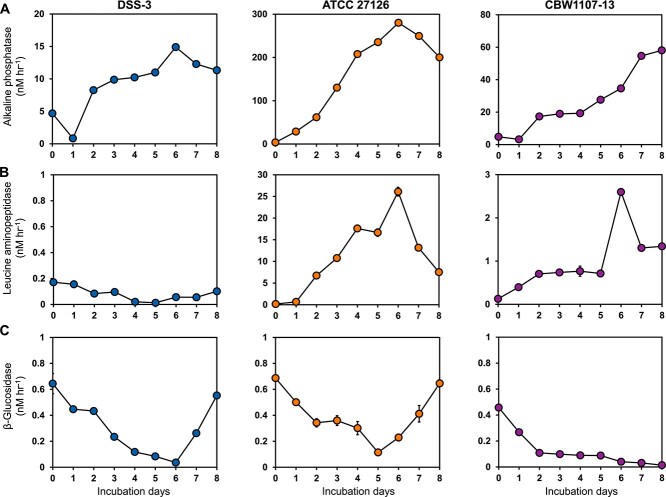
Enzymatic responses of three virion-based bacterial cultures (DSS-3, ATCC 27126 and CBW1107-13). The activity of alkaline phosphatase (A), leucine aminopeptidase (B), and β-glucosidase (C) in each incubation.

Activities of three extracellular enzymes, alkaline phosphatase (APase), leucine aminopeptidase (LAPase), and β-glucosidase (BGase) were measured during bacterial growth in virion mediums. The APase activity was high in all three bacterial cultures (Fig. 2A). The LAPase activity was high in CBW1107-13 and ATCC 27126 cultures, but low in DSS-3 culture (Fig. 2B). The BGase activity in all three cultures remained below 1 nM hr^−1^ (Fig. 2C), indicating that β-glucosidase was not active in the three bacterial cultures. APase catalyzes the regeneration of phosphorus from organophosphate esters, while LAPase is involved in protein degradation [72]. Their substrates align with the chemical composition of viral capsids and chromosomal DNA, which are rich in nitrogen and phosphorus. The fact that APase and LAPase activities increased with bacterial abundance and were significantly inversely correlated with viral abundance (Supplementary Fig. 2) suggests that these bacteria likely employ these extracellular enzymes to degrade viral particles and turn them into bioavailable DOM for bacterial growth. Notably, the low LAPase activity observed in the DSS-3 culture suggests that LAPase is likely not the primary peptidase employed by DSS-3 for degrading virion proteins. A total of 20 peptidase genes and 19 protease genes were predicted in the DSS-3 genome (NC_003911.12), none of which were annotated as LAPase homologs. It is possible that DSS-3 employed peptidase or proteases other than LAPase to degrade virion proteins.

Significant differences in extracellular enzyme activities were observed among the three bacterial cultures. APase and LAPase activities in ATCC 27126 cultures were significantly higher than those in DSS-3 and CBW1107-13 cultures. Specifically, APase activity in ATCC 27126 was 18.8- and 4.8-fold higher than that in DSS-3 and CBW1107-13, respectively, while LAPase activity was 152.5- and 10.0-fold higher, respectively (Fig. 2A and B). The higher extracellular enzyme activities of ATCC 27126 were consistent with findings that *Gammaproteobacteria* serve as major contributors of genes encoding both secreted and extracellular enzymes in marine exoproteomes, as demonstrated across diverse oceanic regions, including the Pacific, Atlantic, and Southern Oceans [73–75]. This also implies that viral particles may serve as important substrates hydrolyzed by gammaproteobacterial extracellular enzymes in the marine environment.

Notably, within each culture, APase activity consistently exceeded LAPase activity. APase activities were 10.7- and 22.4-fold higher than LAPase in ATCC 27126 and CBW1107-13, respectively. This finding appears inconsistent with the nature of viral capsid protein, which primarily requires protease (e.g. LAPase) for degradation. The predominance of APase over LAPase observed in this study may stem from APase’s broader substrate specificity and its dual role in nutrient acquisition. It is reported that APase hydrolyzes commonly occurring phosphoester bonds [76, 77], whereas LAPase primarily targets N-terminal leucine residues in peptides [78, 79]. Moreover, under carbon-limited conditions, bacteria likely upregulate APase activity to degrade organic phosphoric esters, thereby simultaneously liberating phosphate and generating bioavailable organic carbon [72, 80]. Thus, the marked predominance of APase over LAPase across bacterial strains suggests that phosphoester hydrolysis—driven by ecological advantages in nutrient acquisition—may outweigh proteolytic needs in this system, despite the theoretical requirement for LAPase in viral capsid degradation.

### Bacterial utilization of virion DOM

**Figure 3:**
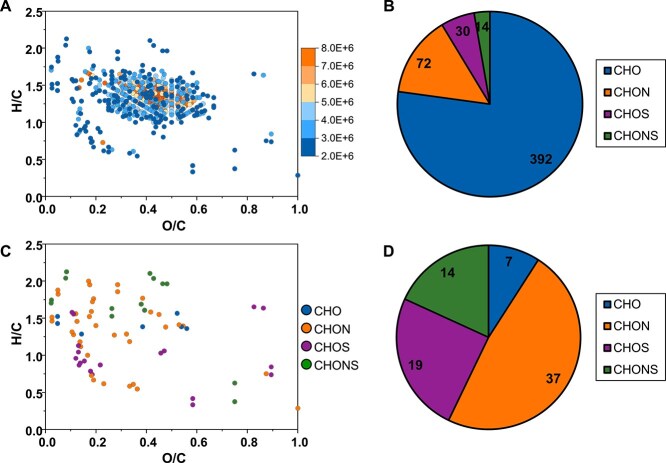
Virion DOM components and their utilization by three bacterial strains. A: van Krevelen diagram of the virion DOM molecules. Color scale indicates the molecular relative intensity. B: Molecular number of virion DOM. C: van Krevelen diagram of the molecular composition of the virion DOM that disappeared in all three bacterial strain cultures. D: Molecular number of virion DOM that disappeared in all three bacterial strain cultures by chemical category.

**Figure 4:**
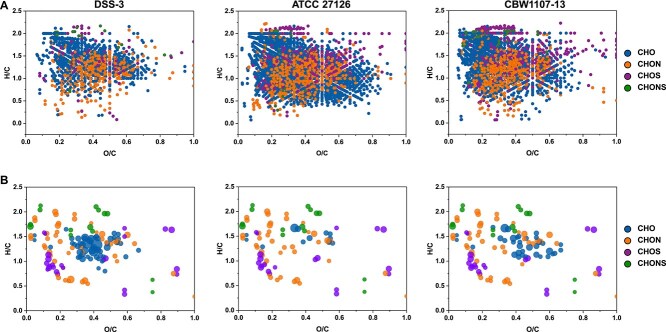
Bacterial transformation and utilization of virion DOM. A: The van Krevelen diagram presents the bacterial culture DOM on day 6. B: The different categories of undetected virion DOM molecules in bacterial cultures. The size of the bubbles represents the relative intensity of the molecules within the virion DOM. The upper three panels illustrate the diverse DOM molecules resulting from the bacterial utilization and transformation of virion DOM. The lower three panels demonstrate the bacterial preference for depleting N- and S-rich virion DOM molecules.

The DOM composition derived from viral particles and the bacterial cultures after incubation were characterized using FT-ICR MS. A total of 508 DOM molecules derived from purified virions were detected (Fig. 3A, Table 1, Supplementary Data 1). Among them, 392 CHO molecules, 72 CHON molecules, 30 CHOS molecules, and 14 CHONS molecules were identified (Fig. 3B and Table 1), accounting for 77.2%, 14.2%, 5.9%, and 2.7% of the total molecules, respectively. Six days after the purified virions were added to bacterial cultures, 77 virion molecules that were initially detected were not detected after incubation with bacteria (Fig. 3C), suggesting that these compounds could be utilized by bacteria. The undetected virion DOM molecules in bacterial cultures were dominated by the CHON-, CHOS-, and CHONS-classes (Fig. 3D), suggesting that bacteria prefer to use nitrogen- and sulfur-rich organic compounds in purified virions. The number of DOM molecules detected in the three bacterial cultures with added virions on day 6 is shown in Fig. 4A (Supplementary Data 1). Notably, 137, 87, and 114 virion DOM molecules undetected in the DSS-3, ATCC 27126, and CBW1107-13 cultures, respectively (Fig. 4B, Table 1). The 77 undetected virion molecules were shared in all three bacterial cultures (Fig. 3C). In terms of uptake of virion DOM molecules, all three bacteria appear to utilize more CHON, CHOS, and CHONS compounds than CHO (Table 1). The FT-ICR MS analysis suggests bacterial utilization of nitrogen- and sulfur-containing molecules derived from virions, supporting the observations that marine viral particles can serve as a DOM source for bacterial growth. Furthermore, the undetected virion DOM molecules represent the fraction that was completely depleted during incubation, thus offering a very conservative estimate of the bacterial utilization. Additionally, the partially depleted or transformed molecules that remain detectable may also be assimilated for bacterial growth.

**Table 1:** Virion DOM molecules that were initially detected but not detected after incubation with bacteria.

Category	Virion DOM molecular no.	Undetected molecular no. (proportion)		
		DSS-3	ATCC 27126	CBW1107-13
CHO	392	52 (13.3%)	12 (3.1%)	35 (8.9%)
CHON	72	47 (65.3%)	41 (56.9%)	44 (61.1%)
CHOS	30	24 (80.0%)	20 (66.7%)	21 (70.0%)
CHONS	14	14 (100%)	14 (100%)	14 (100%)

Notably, the high utilization proportions of CHOS and CHONS molecules by different bacterial taxa suggest that organic sulfur derived from viral particles is highly bioavailable and constitute key elements supporting bacterial growth. This preference for sulfur-containing molecules may stem from bacterial demand for specific sulfur compounds to synthesize sulfur-containing amino acids [81] and cofactor-like coenzymes [82], which are essential for protein synthesis [81] and energy metabolism [83, 84]. Thus, bacterial degradation of virus-derived DOM may represent an important way influencing the oceanic sulfur biogeochemical cycling by converting organic sulfur viral particles into bacterial biomass or inorganic sulfur (e.g. H₂S) [85, 86]. These findings broaden our understanding of virus-mediated elemental cycling.

### Implications for marine elemental cycling

Traditional frameworks for marine viral ecology mainly focused on the “viral shunt”, a process in which the viral lysis of host cells shunts carbon and nutrients flow from higher trophic levels into pools of dissolved and particulate organic matter [4, 5]. Emerging consensus recognizes viral particles themselves as significant elemental reservoirs in the ocean [6, 45]. Findings in this study on bacterial utilization of the phage-derived DOM, along with the recently reported assimilation of coccolithovirus-derived C and N by a coastal bacterial and protist assemblage [51], collectively establish viruses as a dynamic nutrient pool in marine ecosystems. The bacterial utilization of viruses exerts profound impacts on marine food webs and biogenic element cycling through a dual mechanism. First, it directly assimilates virion DOM into bacterial biomass, enhancing secondary production and supplying more resources to higher trophic levels. Second, this process continuously removes viral particles from the environment. It has been reported that a coastal bacterial community removes coccolithoviruses at a rate 33% higher than abiotic controls [51]. The resulting reduction in infectious virus abundance lowers the viral lysis of host cells. Consequently, this effect weakens the viral shunt and enhances biomass transfer to higher trophic levels in the microbial loop, which directly alters the carbon flux in marine ecosystems [87].

Moreover, our findings revealed that extracellular enzymes may play a central role in the bacterial utilization of virion DOM. Extracellular enzymes are thought to initiate bacterial remineralization of organic matter in the marine environment by hydrolyzing high-molecular-weight compounds into small substrates [88]. High extracellular enzyme activities, especially those associated with viral degradation (APase and LAPase), are consistently observed across diverse marine ecosystems [80, 89–97] and play a major role in the virus decay in the marine environment [52, 98]. It is estimated that extracellular enzyme activities contribute ~20% of maximal viral decay in coastal surface seawater [98] and account for 14.2%–44.0% of the virus decomposition in the surface deep-sea sediments across different oceanic regions [52], far exceeding the impact of abiotic factors. The prevalence of extracellular enzymes and their high efficacy in virus decomposition indicate that bacterial assimilation of virion DOM is a widespread phenomenon, which substantially influences elemental cycling in the global ocean.

## Conclusion

This study proved that viral particles can support the growth of various non-host heterotrophic bacteria. The degradation of viral particles by bacteria could be mediated by secreting extracellular enzymes, particularly APase and LAPase, facilitating the bacterial acquisition of phosphorus and nitrogen from viral particles. Notably, in addition to nitrogen-containing components, different bacteria also efficiently utilize sulfur-containing molecules in virion DOM. Our observations demonstrate the ecological functions of marine viruses as a nutrient source for bacteria and their impacts on biogeochemical element cycling of the marine ecosystem. This study focusing on a single phage and limited bacterial strains, represents a simplified scenario of the marine ecosystem. Future research on the degradation of diverse viruses by complex natural bacterial communities are demanded to fully uncover the mechanisms and quantify the broader impact of this process on nutrient fluxes in marine ecosystems.

## Supplementary Material

Supplementary_Information_ycag056

Supplementary_Data_1_Molecular_compositions_in_this_study_ycag056

## Data Availability

All data generated or analyzed during this study are included in this published article and its supplementary information files.
