# Supplementary material for: Marine bacteria degrade viral particles as a source of nitrogen, phosphorus, and sulfur-rich dissolved organic matter
Source: ISME Commun. 2026 Mar 9;6(1):ycag056. doi: 10.1093/ismeco/ycag056 (PMC13064650; doi:10.1093/ismeco/ycag056)
Supplement: Supplementary_Information_ycag056 [file supplementary_information_ycag056.docx]

**Supplementary Information for**

**Marine bacteria degrade viral particles as a source of nitrogen, phosphorus, and sulfur-rich dissolved organic matter**

Hongcong Man^1,2^, Xiaojue Li^1,2^, Jihua Liu^1,2^, Chen He^3^, Quan Shi^3^, Xilin Xiao^4^, Wei Wei^5^, Feng Chen^6,*^, Yongle Xu^1,2,*^

^1^Institute of Marine Science and Technology, Shandong University, Qingdao 266237, China

^2^Shandong Key Laboratory of Intelligent Marine Engineering Geology, Environment and Equipment, Qingdao 266237, China

^3^State Key Laboratory of Heavy Oil Processing, China University of Petroleum, Beijing 102249, China

^4^Innovation Research Center for Carbon Neutralization, Xiamen University, Xiamen 361002, China

^5^Hubei Key Laboratory of Microbial Transformation and Regulation of Biogenic Elements in the Middle Reaches of the Yangtze River, State Key Laboratory of Green and Efficient Development of Phosphorus Resources, School of Environmental Ecology and Biological Engineering, Wuhan Institute of Technology, Wuhan 430205, China

^6^Institute of Marine and Environmental Technology, University of Maryland Center for Environmental Science, Baltimore, MD 21202, United States

^*^Correspondence: [xuyongle@sdu.edu.cn](mailto:xuyongle@sdu.edu.cn) (Y.L. Xu) and [chenf@umces.edu](mailto:chenf@umces.edu) (F. Chen)

**This file contains:**

Supplementary Figures 1–3

Description of Supplementary Data 1

**Other Supplementary Material includes the following:**

Supplementary Data 1


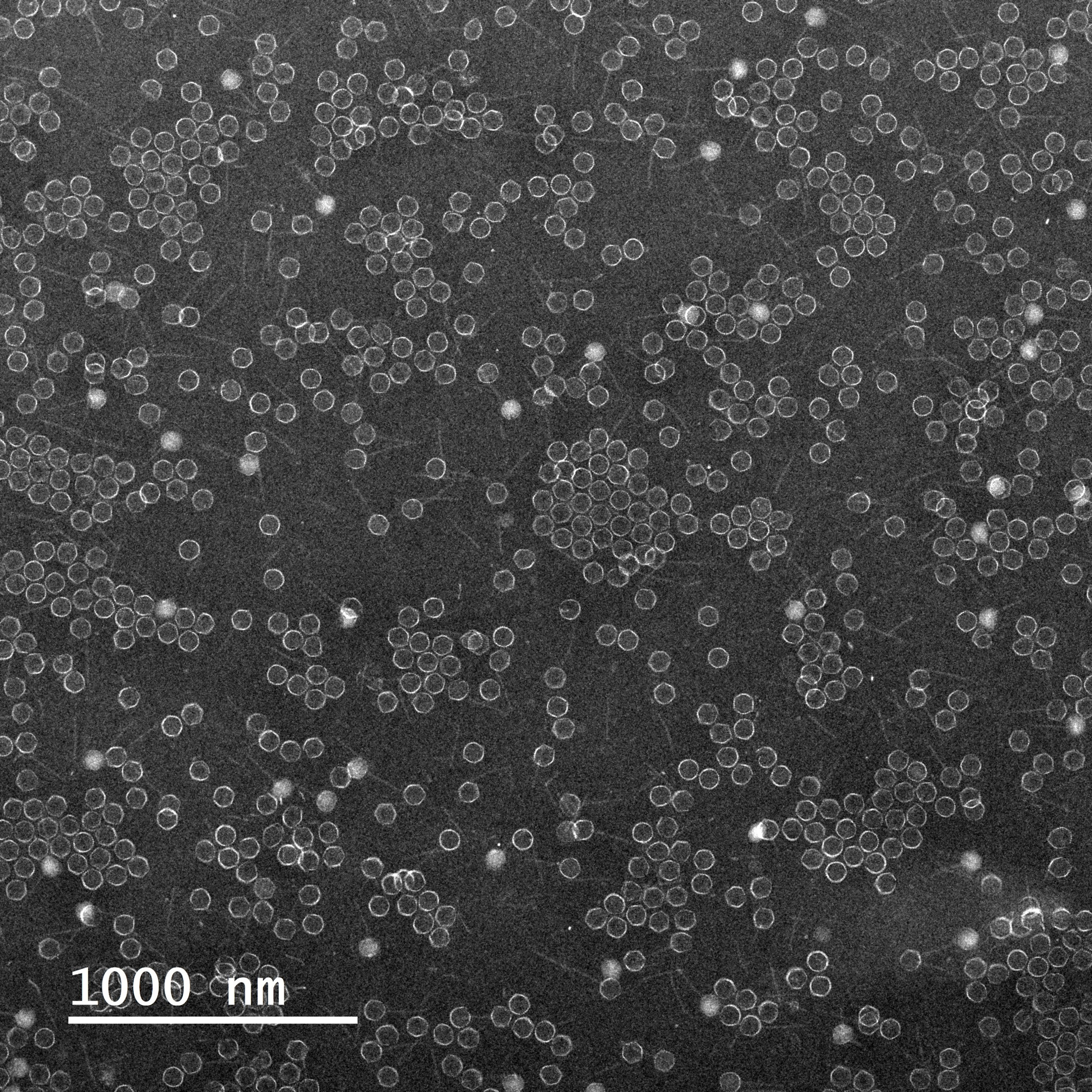


Supplementary Figure 1. Highly purified viral particles used for bacterial cultivation as DOM substrate.


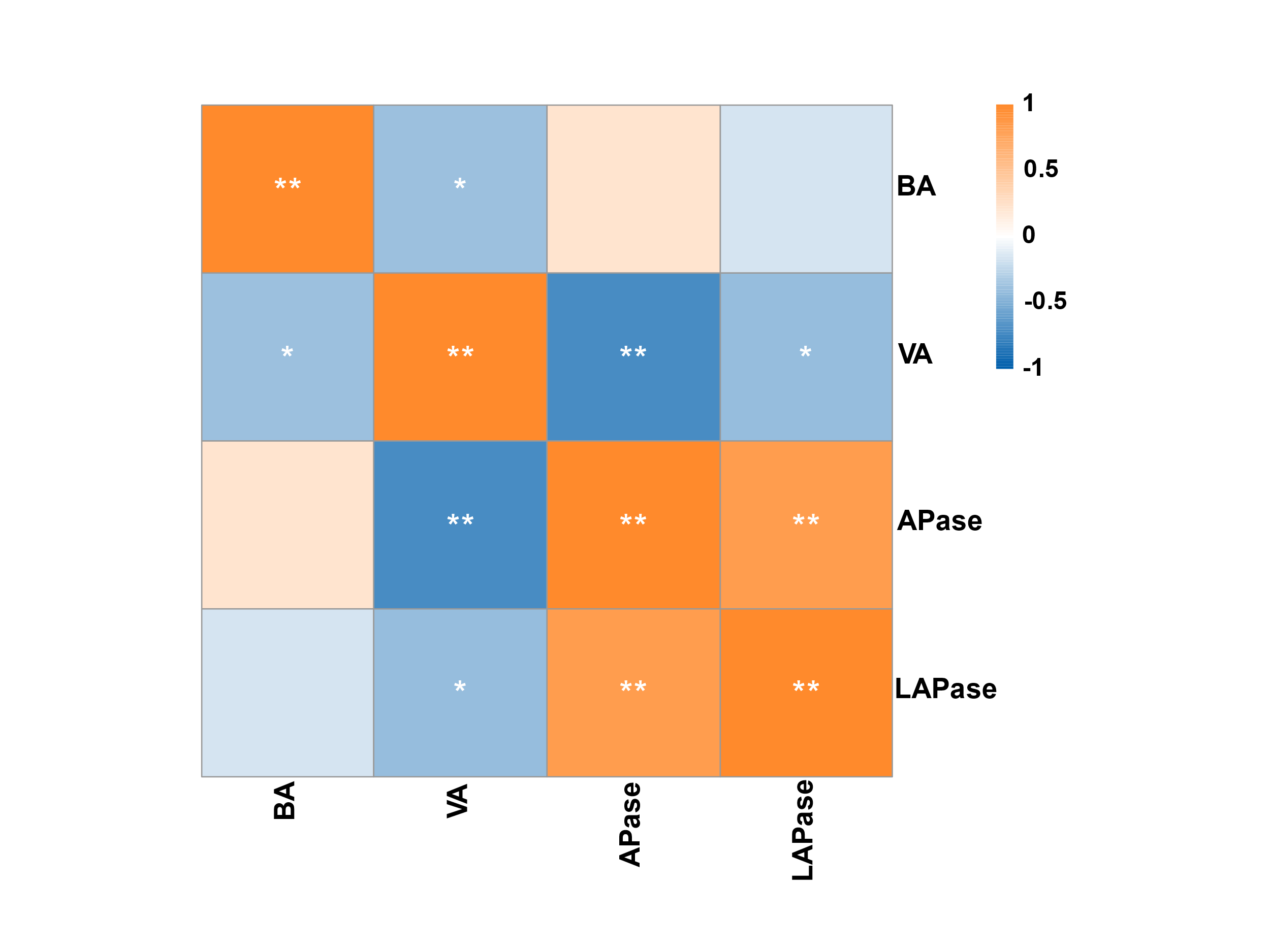


Supplementary Figure 2. Clustering Spearman correlation heatmaps for the microbial abundances (BA: bacterial abundance, VA: viral abundance) and enzymic activities of APase and LAPase. Level of significance: *P < 0.05; **P < 0.01.


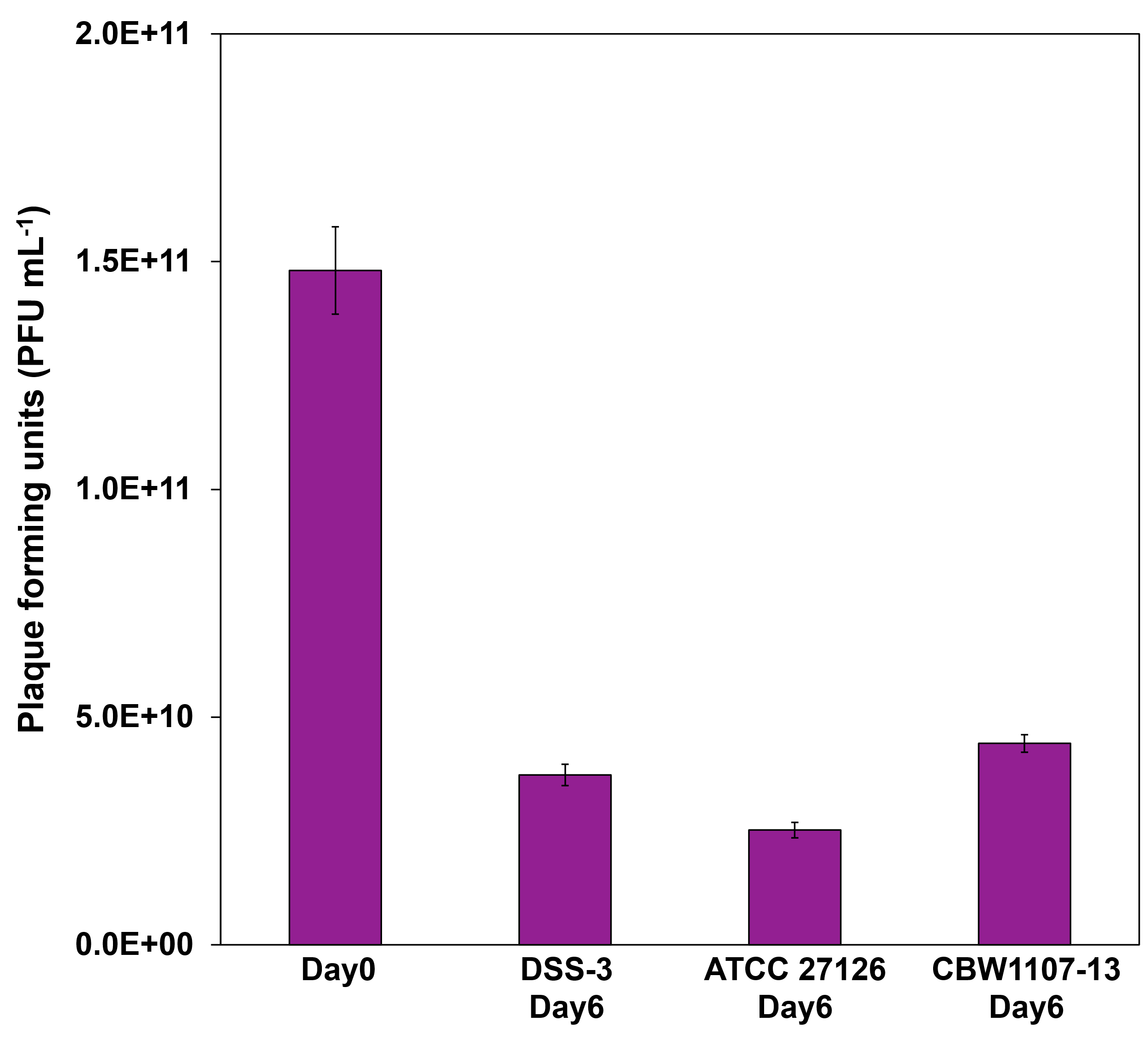


Supplementary Figure 3. PFU variations of RDCBphi1 in three virion-based bacterial cultures.

**Description of Supplementary Data File**

Supplementary Data 1: Molecular compositions in this study
